# Genetic loci regulating cadmium content in rice grains

**DOI:** 10.1007/s10681-020-02752-1

**Published:** 2021-02-10

**Authors:** Gareth J. Norton, Anthony Travis, Panthita Ruang-areerate, Graeme W. Nicol, Ayotunde A. Adeosun, Mahmud Hossain, M. Rafiq Islam, Alex Douglas, Adam H. Price

**Affiliations:** 1 School of Biological Sciences, University of Aberdeen, Aberdeen, AB24 3UU UK; 2 National Omics Center, National Science and Technology Development Agency (NSTDA), Pathum Thani, 12120 Thailand; 3 Laboratoire Ampère, École Centrale de Lyon, Université de Lyon, 36 avenue Guy de Collongue, 69134 Ecully Cedex, France; 4 Department of Soil Science, Bangladesh Agricultural University, Mymensingh, Bangladesh

**Keywords:** Rice, Cadmium, Genome wide association genetics, Alternate wetting and drying

## Abstract

It has been estimated that up to 90% of human exposure to cadmium is through food, and that cadmium within rice grains can be a major contributor to that dietary source. In this study genome wide association mapping was conducted on the Bengal and Assam Aus Panel (BAAP) of rice to identify quantitative trait loci and candidate genes for lowering grain cadmium. Field experiments were conducted over two years under two different irrigation systems: continually flooded and alternate wetting and drying (AWD). There was significant effects of water treatment, genotype, and genotype by water treatment interaction. Importantly, AWD increased grain cadmium, on average, by 49.6% and 108.8% in year 1 and 2 respectively. There was between 4.6 and 28 fold variation in cadmium concentration. A total of 58 QTLs were detected but no loci are clearly specific to one water regime despite approximately 20% of variation attributable to genotype by water regime interaction. A number of QTLs were consistent across most water treatments and years. These included QTLs on chromosome 7 (7.23–7.61, 8.93–9.04, and 29.12–29.14 Mbp), chromosome 5 (8.66–8.72 Mbp), and chromosome 9 (11.46–11.64 Mbp). Further analysis of the loci on chromosome 7 (8.93–9.04 Mbp), identified the candidate gene *OsNRAMP1*, where cultivars with a deletion upstream of the gene had higher concentrations of cadmium compared to the cultivars that did not have the deletion. The distribution of alleles within the BAAP suggest this QTL is easily detected in this population because it is composed of *aus* cultivars. Local genome cluster analysis suggest high Cd alleles are uncommon, but should be avoided in breeding.

**Supplementary Information:**

The online version contains supplementary material available at (10.1007/s10681-020-02752-1).

## Introduction

Cadmium in rice grains has been highlighted as an issue, specifically for people that consume large quantities of rice, with a proportion of people in Bangladesh and Sri Lanka consuming rice with unsafe amounts of cadmium (Meharg et al. 2013). It is estimated that for non-smokers 90% of cadmium exposure is through food, while for smokers it is 50%. (FAO/WHO 2001; UNEP 2008). Cadmium can accumulate in several organs, including the liver and kidneys, and in humans can result in itai-itai disease (Kobayashi et al. 2009).

One of the main driving factors for cadmium uptake by plants is its availability in soils. Under anaerobic soil management, for example cultivation of rice in a flooded field, cadmium is present as immobile cadmium sulphide (Khaokaew et al. 2011; Hashimoto and Yamaguchi 2013) or cadmium carbonates (Khaokaew et al. 2011). However, in aerobic soils the cadmium is present as the Cd^2+^ ion. It is the Cd^2+^ that is readily taken up by plants. As freshwater for rice irrigation is considered unsustainable in some parts of the word, rice cultivation under different water management strategies may result in an increase in cadmium availability to rice plants. It has been demonstrated that cadmium concentration in rice grains increases when plants are grown under the water saving irrigation practice of alternate wetting and drying (AWD) (Norton et al. 2017a; Li et al. 2019). AWD is an irrigation practise where the standing water in the fields is allowed to drain until either a specific soil water potential is reached or the water height is at a specific level below the soil surface (normally between 10 and 20 cm) (Lampayan et al. 2015). Grain cadmium concentration has been observed to increase by 28–67% in AWD-grown plants compared to plants grown under flooded irrigation practises (Norton et al. 2017a, b).

The uptake, transport, and accumulation of cadmium in plants has been the focus of numerous studies. Key genes involved in the accumulation of cadmium have been reported in rice. For example, *OsHMA3* and *OsHMA2*, which are P1B-type Heavy Metal ATPases, have been demonstrated to be involved in cadmium accumulation. *OsHMA3* is involved in vacuolar sequestration of cadmium (Ueno et al. 2010; Sasaki et al. 2014) while *OsHMA2* facilitates xylem loading of cadmium (Satoh-Nagasawa et al. 2012; Takahashi et al. 2012a). Another key family of genes involved in cadmium accumulation in plants is the Natural Resistance Associated Macrophage Proteins (NRAMP). *OsNRAMP1*, *OsNRAMP2*, and *OsNRAMP5* have been shown to be involved in cadmium transport in rice (Sasaki et al. 2012; Takahashi et al. 2011; Zhao et al. 2018). Other genes involved in uptake and transport include the Low Cadmium gene *OsLCD* (Uraguchi et al. 2011; Shimo et al. 2011), the ATP-binding cassette *ABCG43* (Oda et al. 2011), *CAL1* (Luo et al. 2018), the rice iron-regulated transporters (*OsIRT*) 1 and 2 (Nakanishi et-al. 2006), the Low Affinity Cation Transporter *OsLCT 1* (Uraguchi et al. 2011), and *OsZIP1* (Nakanishi et al. 2006). Genes that function as cadmium transporters also function as transporters of other elements (e.g. iron, zinc, and manganese) (Yoneyama et al. 2015), including *OsIRT 1* and *2* (Bughio et al. 2002; Ishimaru et al. 2006), *OsNRAMP5* (Ishimaru et al. 2012), and *OsHMA3* (Ueno et al. 2010; Miyadate et al. 2011).

Mutant, knockout, and overexpression studies targeting the above genes have been shown to alter the accumulation of cadmium in rice grains; examples include studies by Ishikawa et al. (2012), Ueno et al. (2010), and Miyadate et al. (2011). However, these can produce other undesired impacts in terms of alteration of the accumulation of other elements. In a study where *OsNRAMP5* was mutated, it resulted in rice grains with a nearly undetectable concentration of cadmium (Ishikawa et al. 2012), but there was also a decrease in manganese of more than tenfold within the shoots and three fold in the grains compared to the control plants (Ishikawa et al. 2012). In another study where OsNRAMP5 was knocked out it resulted in plants that accumulate less manganese in the roots and less manganese and iron in the shoots (Ishimaru et al. 2012).

There are significant genotypic differences in grain cadmium concentration in rice. These genotypic differences have enabled the identification of quantitative trait loci (QTL) for cadmium concentration in rice plants. These studies have found numerous QTLs for cadmium concentration in the roots, shoots, and grains of rice plants. Recent advances in genetic mapping in plants have meant that genome wide association (GWA) studies are feasible in rice (Zhao et al. 2011). To date, five GWA studies has been conducted in rice for grain cadmium all in the last two years (Zhang et al. 2018; Zhao et al. 2018; Lui et al. 2019; Tan et al. 2020; Yan et al. 2019) using different populations mostly comprised of *indica* or *japonica* subpopulations or a mixture of both. In respective order they identified 61, 20, 17, 19 and 11 QTLs for grain Cd in rice.

The Bengal and Assam Aus Panel (BAAP), which has 2 million SNPs for GWA mapping, has been specifically created to assess the genetic diversity within the *aus* subpopulation of rice, and allow traits influenced by flowering time to be better studied (see Norton et al. 2018). To emphasise the features of the BAAP; (1) population structure, which is a hindrance to association studies, is reduced (but not eliminated) in the BAAP by using one subpopulation; (2) the *aus* subpopulation is recognised to be understudied (Kim et al. 2016) yet contains wide trait variation for abiotic stress resistance, (3) the influence of weather or soil conditions at flowering time on grain-related traits has been minimised by producing a population with a small flowering window (Norton et al. 2018); (4) the BAAP has been successfully used to map QTLs for grain arsenic that would not have been detected in *indica*, *japonica* or a mixed *indica/japonica* population (Norton et al. 2019).

This study utilises the Bangladesh and Assam Aus Panel (BAAP) (Norton et al. 2018), grown in the field in two consecutive years under two water management strategies—continually flooded (CF) conditions and AWD. The objectives are to identify QTLs and genes that might be useful in breeding lower grain Cd, and to determine if AWD influences the QTLs such that breeding might need to be different for different irrigation regimes.

## Methods

### Population and field screen

The population used in this study was the Bengal and Assam Aus Panel (BAAP) (Norton et al. 2018). The population consists of 266 landraces identified as belonging to the *aus* subpopulation, in addition to the OryzaSNP panel (McNally et al. 2009) and a number of elite Bangladeshi cultivars (Norton et al. 2018). The 266 *aus* cultivars from the population have been sequenced, and a 2 million SNP database constructed (Norton et al. 2018).

The BAAP was grown in the field in Mymensingh, Bangladesh in the Boro (dry season) of 2013 and 2014. The population was screened under both AWD and continuously flooded (CF). Full details of the field screening is given in Norton et al. (2017a) and Norton et al. (2018). In both years the seeds were initially grown in a nursery bed and transplanted to the field plot after 44 days in 2013 and 51 days in 2014. The plants were transplanted into eight experimental plots (four control and four AWD), two plants per hill with a distance of 20 cm between each hill in a row and a 20 cm distance between each row of 2 m length. Rice accessions were planted in single rows, with a check cultivar BRRI Dhan 28 transplanted into each alternate row. After transplanting the plots were flooded. For the four CF plots the surface water was kept at a depth of between 2 and 5 cm above the soil surface during the vegetative stage and reproductive stage (13th April 2013). For the four AWD plots plastic perforated tubes (pani pipe) were placed across the plots to monitor the water depth. The aim was to allow water to drain/percolate naturally from the AWD plots until the average depth of the water was 15 cm below the soil surface. At that point the plots were irrigated to bring the water depth to between 2 and 5 cm above the soil surface (Norton et al. 2017a). AWD was applied only from 14 days after transplanting until flowering. Once the cultivars had flowered and the grains matured, the grain and shoots from every cultivar was hand harvested from the six central hills of each row. This resulted in one sample for each of the four AWD and one sample for each of the four control plots.

### Cadmium analysis

Grain cadmium concentration was determined as described in Norton et al. (2017a). Briefly, rice grains were de-husked and oven dried, followed by microwave digestion with concentrated nitric acid and hydrogen peroxide as described in Norton et al. (2012). Total cadmium analysis was performed by inductively coupled plasma–mass spectroscopy (ICP–MS). Trace element grade reagents were used for all digests, and for quality control replicates of certified reference material rice flour [NIST 1568b]) were used; blanks were also included. All samples and standards contained 10 µg L^−1^ indium as the internal standard.

### Genome-wide association (GWA) mapping

Prior to GWA mapping the mean grain cadmium concentration for each treatment and year were calculated only for accessions with values for at least three replicates. GWA mapping was conducted using “PIQUE” (Parallel Identification of QTL’s Using EMMAX, https://github.com/tony-travis/PIQUE) to pre-process genotype and phenotype data and then run association analysis for each phenotype in parallel using EMMAX (Kang et al. 2010) as described in Norton et al. (2018).

### Clump analysis of significant SNPs

SNPs were binned together into peaks using a sliding window based on the decay of linkage disequilibrium (LD) using the PLINK (Purcell et al. 2007) command—clumpp1 0.000001–clump-p2 0.00001–clump-r2 0.3–clump-kb 243. Note the command-clumpp1 0.000001 indicates a very high *P* value threshold (*P* < 1 × 10^–6^) was employed only to keep the number of detected QTLs manageable. For every SNP with *P* < 0.000001, pairwise r^2^-values were calculated between surrounding SNPs that fell within 243 kb; any two SNPs meeting this criteria that also shared an r^2^ ≥ 0.3 were clumped into bins. Binning was done for each experiment. Clumps with less than two significant SNPs (*P* < 0.00001) were discarded. The LD (Linkage Disequilibrium) decay value of 243 kb was selected as this is the average LD decay for the population (Norton et al. 2018).

### Determination of local linkage disequilibrium (LD)

Local LD decay was estimated as r^2^ (VanLiere and Rosenberg 2008) using PLINK (Purcell et al. 2007). LD decay within a 1 Mb genomic region centred on the most significant SNP was determined by calculating all pairwise r^2^ values between SNPs and binning LD decay according to distance.

### Assignment of genotype clusters within candidate regions

A cluster analysis was conducted on the SNPs identified in the QTL defined by clump. Specifically, an alignment of 2002 SNP positions from 260 genotypes was analysed with a simple pairwise comparison distance approach using MEGA7 (Kumar et al. 2016). Each sequence was compared in a pairwise manner with individual nucleotide positions containing a degeneracy (i.e. where different bases were present on two alleles) excluded from the analysis on a pairwise basis. Distances were calculated using the number of differences method (Nei and Kumar 2000) and cladograms constructed using the neighbour-Joining method (Saitou and Nei 1987). Bootstrap support was calculated from 100 replicates (Felsenstein. 1985) and genotypes clustered based on a 95% node support value. Clusters with less than 5 accessions are not reported.

### Determination of deletion on chromosome 7 for each accession

As the accessions sequenced in this study have only been sequenced to an average depth of 6.5 × the alignment of each individual accession to the reference sequence can lead to errors in base calling. Therefore, an approach focussed on sequencing density, rather than base calling, was performed to evaluate if accessions had deletion upstream of LOC_Os07g15370. The deletion upstream of LOC_Os07g15370 was identified in the 3000 genome dataset (Wang et al. 2018), starting at genome position 8,964,600 bp on chromosome 7 and ending at 8,964,988 bp (total of 388 bp). The number of sequences aligned to the 500 bp regions flanking (upstream and downstream) of the deletion and the deletion for each accession was determined. The ratio (number of counts for the deletion divided by 388/number of counts in the flanking regions divided by 1000) was used to identify accessions with a deletion and those without.

### Statistical analysis

All statistical analyses were performed using the statistical software Minitab v.17 (State College PA) and SigmaPlot v.13 (Systat Software Inc., CA) and significance reported at alpha < 0.05. Analysis of variance across treatments in each year was only conducted on those cultivars present in both treatments (n = 218 in year 1 and 207 in year 2).

## Results and discussion

### Grain cadmium concentration

**Fig. 1:**
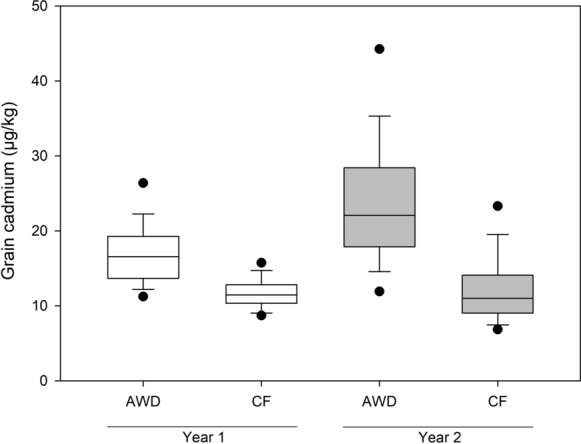
Difference between the accessions in the BAAP grown under AWD or CF field management in year 1 and year 2

The mean grain cadmium concentrations recorded for each cultivar in this study are reported in supplementary Table 1. Grain cadmium concentration was significantly different between the plants grown under AWD and CF in both years (Fig. 1, Table 1). AWD increased grain cadmium, on average by 49.6% and 108.8% in year 1 and 2 respectively, compared to the plants grown under CF (Fig. 1, Table 2). Across all experiments there were genotypic differences for grain cadmium, with genotypic differences explaining between 47.5 and 84.1% of the variation observed in the grain cadmium phenotype depending on treatment and year (Table 1). In both years, highly significant genotype by treatment interactions were observed for grain cadmium concentration, explaining 19.2% and 23.7% of the variation (Table 1).

**Table 1:** Two-way ANOVA results (F-values) for treatment, genotype and the treatment × genotype interaction for the grain cadmium concentrations across 2013 and 2014

Year (n)	Trait	2-way ANOVA F-value		
		Treatment	Genotype	Treatment × genotype interaction
2013 (218)	Grain Cd	817 (10.8%)	13.4 (46.9%)	6.95 (23.7%)
2014 (207)	Grain Cd	545 (10.3%)	10.57 (45.7%)	4.40 (19.2%)

Numbers in brackets are the proportion of the variation explain by that factor. All factors are significant at *P* < 0.001

**Table 2:** Descriptive statistics for grain cadmium concentration measured in 2013 and 2014 for the BAAP accessions grown under AWD and CF

Year	Treatment	Grain Cd (µg/kg)					
		N	Min	Max	Median	Mean	SD
2013	AWD	243	9.6	142.7	16.6	17.8	10.2
	CF	226	7.0	32.1	11.5	11.9	2.8
2014	AWD	220	8.9	251.1	22.1	26.1	22.2
	CF	209	5.2	57.1	11.0	12.5	6.1

**Fig. 2:**
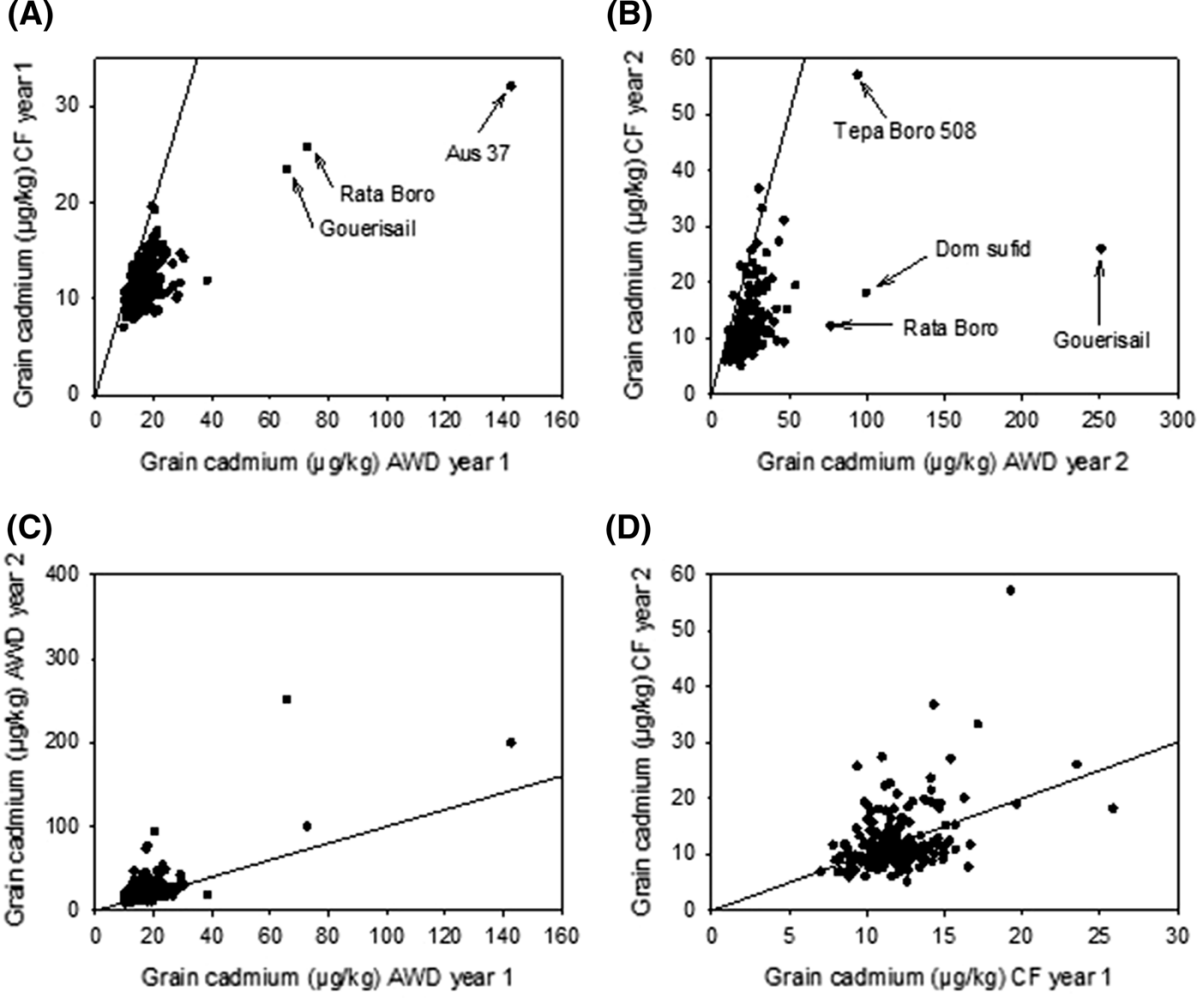
Correlation between grain cadmium concentration for the BAAP grown under; **a** AWD and CF in year 1, **b** AWD and CF in year 2, **c** AWD in year 1 and AWD in year 2, **d** CF in year 1 and CF in year 2. The line on each graph represents the 1:1 line

It has been widely reported that AWD alters the accumulation of a range of elements in rice grains, including cadmium (Norton et al. 2017a; Chou et al. 2016; Linquist et al. 2015; Wang et al. 2014; Yang et al. 2009). For a small sub sample of this population (22 accessions of which 19 are aus and used in the GWAS reported here) the cadmium data reported has been published along-side 16 other grain elements and with results from one additional field site (Norton et al. 2017b). The increased number of accessions reported here has increased the range of grain cadmium concentrations observed in the accessions under both AWD and CF (Table 2). When comparing the relationships of grain cadmium for the cultivars grown under the two different water treatments it is clear that there are a number of cultivars that accumulate high concentrations of cadmium under AWD compared to when grown under CF (Fig. 2). In year 1 these included Aus 37, Rata Boro, and Gouerisail, which accumulated 5.3, 2.8, and 3.1 fold higher cadmium under AWD compared to CF (Fig. 2a). In year 2, Rata Boro, Gouerisail, and Dom Sulfid accumulated 5.5, 5.1, and 4.3 fold higher cadmium under AWD (Fig. 2b). It should be noted that in year 2 Aus 37 had the second highest grain cadmium of the plants grown under AWD in year 1, however it is not presented in Fig. 2, as it was not grown in the CF treatment in year 2. The cultivar Tepa Boro 508 is highlighted in Fig. 2b because it had very high grain cadmium concentration in both AWD and CF in year 2, being three-four times higher than the concentrations in year 1 (Supplementary Table 1). Eight cultivars were consistently found to have low grain cadmium concentrations (Table 3), with ASSAM 4(BORO) and KALIBORO 26 being in the lowest 10% for grain cadmium across all experiments. The observed significant genotype by treatment interaction for grain cadmium (Table 1) has implications for selective breeding for low cadmium cultivars, as these results suggest that different accessions accumulate cadmium through different genetic mechanisms under different water management regimes. In contrast, when exploring other traits, for example grain yield (Norton et al. 2018) and grain arsenic concentration (Norton et al. 2019), it was observed that there would be no specific requirement to breed for these traits under AWD because genotype by treatment interaction was minimal. This is not true for grain cadmium.

**Table 3:** Cultivars with consistently low grain cadmium in three or four of the experiments

BAAP Id	Cultivar name	Year 1		Year 2	
		AWD	CF	AWD	CF
1	ASSAM 4(BORO)	X	X	X	X
74	AUS 364	X	X		X
121	KHAILAORGOABEZ		X	X	X
136	KALIBORO 26	X	X	X	X
151	KALO BIRA	X	X		X
222	Jamir	X		X	X
243	Gobir sail	X	X	X	
265	P 79	X	X	X	

An X indicates that that cultivar was in the lowest 10% for grain cadmium concentration in that experiment

The population used in this study is made up of rice accessions from the *aus* subpopulation of rice (Travis et al. 2015; Norton et al. 2018) and therefore does not have the high degree of stratification of some GWA mapping panels, which have representatives from across the different rice subgroups (Zhao et al. 2011). However, there is a degree of structure to the population, with five discernible *aus* subgroups (Norton et al. 2018). There is a significant difference in grain cadmium concentration between these different subgroups for AWD (*P* = 0.027, df = 4, F = 2.88) and CF (*P* < 0.001, df = 4, F = 5.87) in year 2 (Supplementary Fig. 1), accounting for 7.2% and 18.1% of the observed variation. In year 1 there was no significant difference in grain cadmium for the accessions belonging to the different subgroups. Generally, *aus* subgroup 3 has lower grain cadmium concentrations while subgroups 2 and 4 have higher concentrations.

### Cadmium GWA mapping

**Fig. 3:**
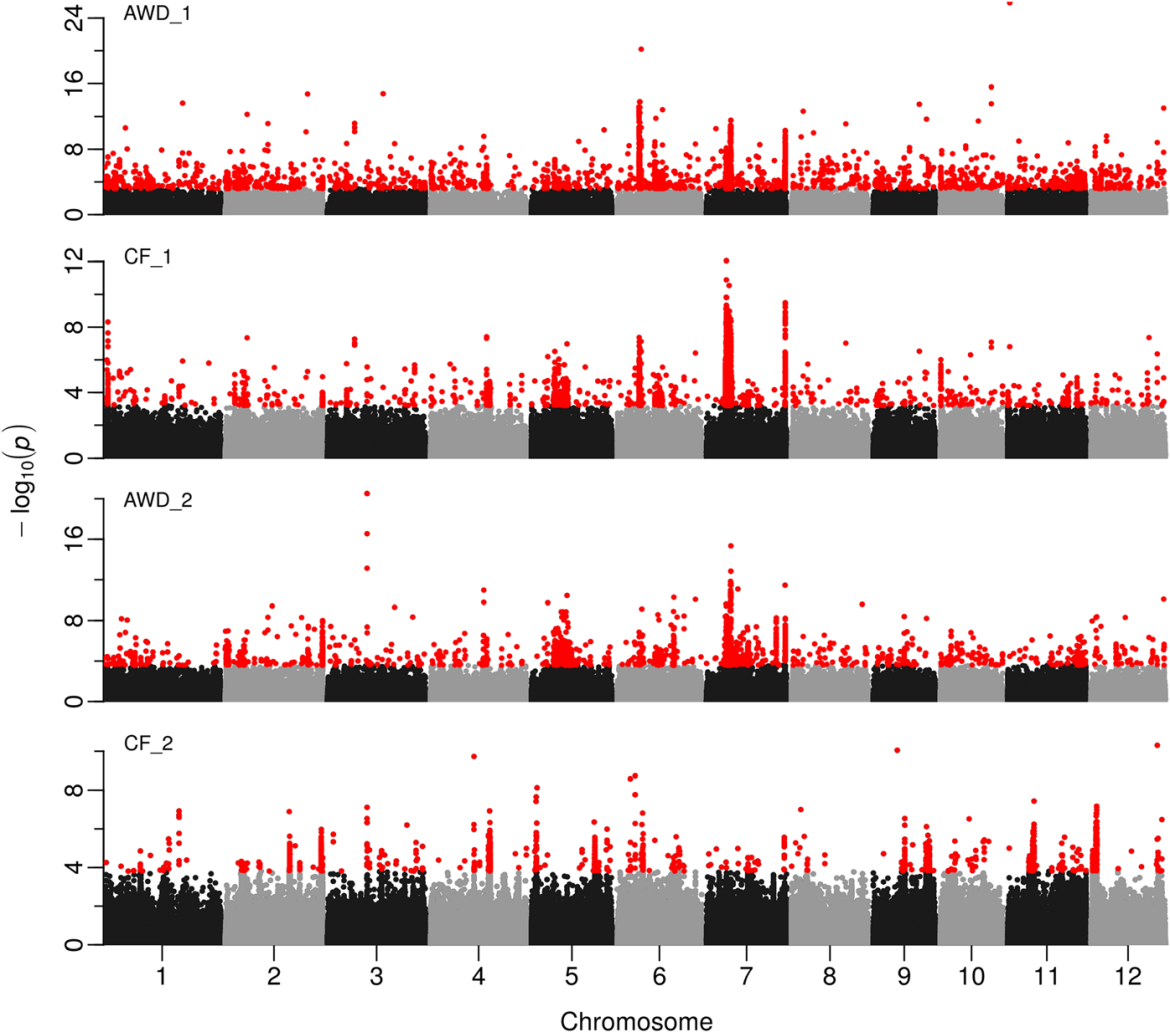
Manhattan plots from GWA mapping of grain cadmium in field experiments under AWD (Alternate Wetting and Drying) and CF (Continuous Flooding) over years 1 and 2. A guide-line in blue is shown at − log10(0.0001) = 4. Benjamini–Hochberg adjusted probabilities > 0.1 are highlighted in red. (Color figure online)

SNP-trait associations were detected for grain cadmium content across the two years of the experiment (Fig. 3). Clump was used to group them for each trait, revealing 31 groups for AWD1, 24 for CF1, 35 for AWD2 and 13 for CF2 (Supplementary Table 2). These were then combined and further grouped together as a single QTL if any group was within 243 kb (the global LD for BAAP), revealing a total of 58 QTLs (Supplementary Table 2). Most of these (41) were detected only in one trait (9 for AWD1, 2 for CF1, 22 for AWD2 and 8 for CF2) while 12 were detected in two traits and five were detected in three traits (in each AWD1, CF1 and AWD2). None were detected in all traits. However, it must be noted that a high threshold was used for calling SNP-trait associations (*P* < 1 × 10^–6^) which will probably introduce type II error. A probable example can be seen at the bottom of chromosome 7 where all traits have a peak but it is called only for three traits because for CF2 the *P* value was 9.4 × 10^–5^ (for SNP at 29.15 Mbp) and therefore not above the threshold used. Even some of the single trait QTLs contained large numbers of SNPs with *P* < 0.0001, (up to 2694 SNPs for AWD2 at 8.17 on chromosome 5). AWD2 was notable for having a very large number of single trait QTLs, while CF2 was notable for having by far the fewest QTLs, only 5 of which were common to another trait, and none common to two other traits. Examining Fig. 3 also suggests CF2 is different to the other three traits. Previous GWAS studies, using other populations also detected many significant loci for grain cadmium and some co-localize with QTLs in the BAAP. A genetic map of the current results shown with previous studies is given in supplementary Fig. 2. To summarise co-localisation (see supplementary Table 3), Zhang et al. (2018) reported 61 QTLs of which 8 are common with the BAAP, Zhao et al. (2018) reported 20 QTLs of which 4 are common with the BAAP, Lui et al. (2019) reported 17 QTLs of which 1 is common with the BAAP, Tan et al. (2020) reported 19 QTLs of which 3 are common with the BAAP while Yan et al. (2019) reported 11 QTLs of which 2 are common with the BAAP. A striking feature of current and previous Cd QTLs presented in supplementary Fig. 2 is the high number of QTLs, their distribution and relatively small proportion of loci that are common between studies. What distinguishes this study is that here we have detected loci across multiple treatments and years. This has notable benefits when investigating these QTLs further, primarily that this treatment/year stability is essential when breeding rice with reduced cadmium as loci could be of use in a wider array of environments and under a diverse range of management approaches. Five loci are discussed, where co-localised QTLs are detected in at least three of the four of the experiments.

### QTL on chromosome 7 between 7.23 and 7.61 Mbp

**Fig. 4:**
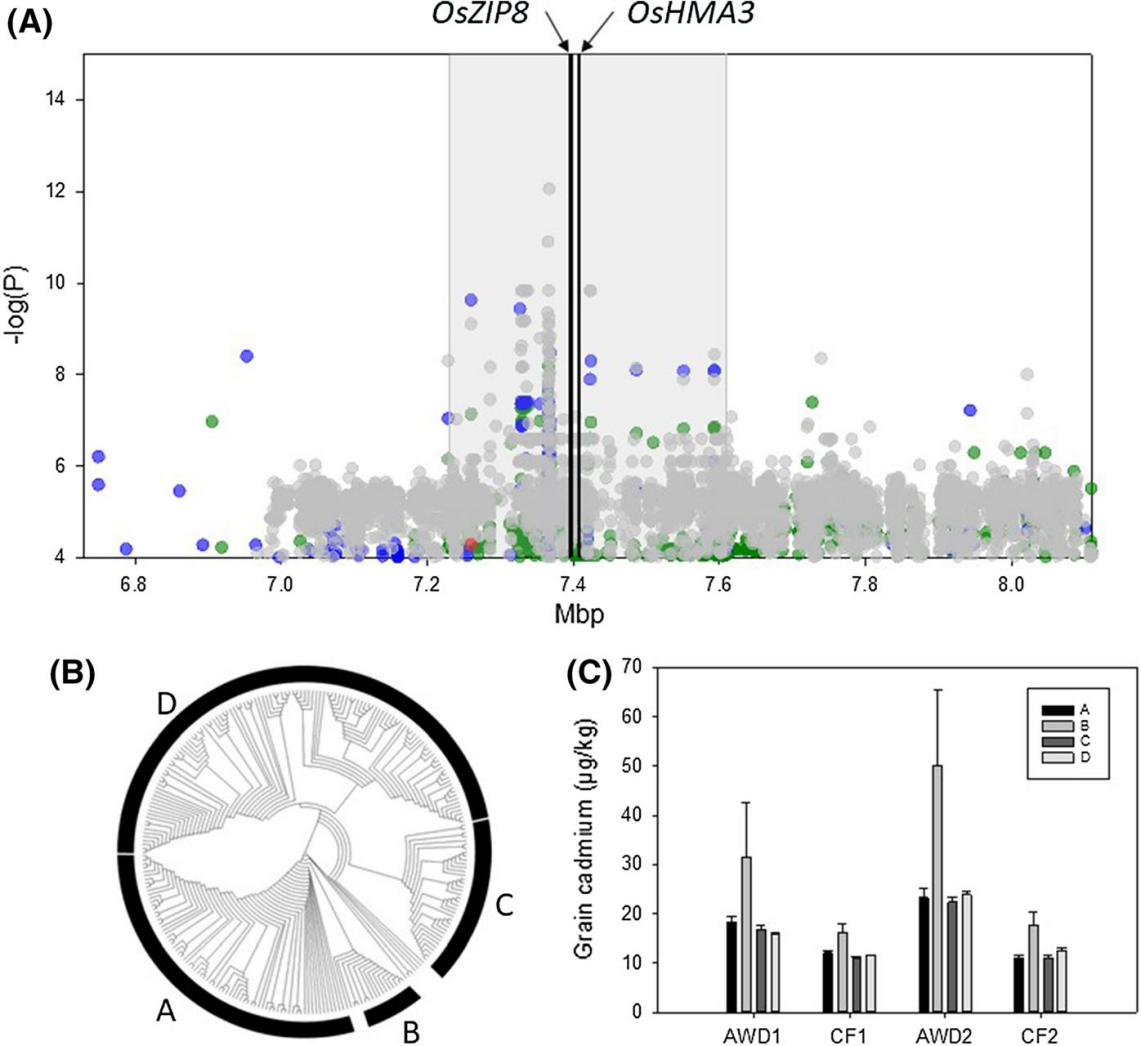
Significant associations for cadmium traits on chromosome 7 (6.7–8.2 Mb). **a** SNPs associated with the cadmium phenotypes; green symbols = AWD year 1, blue symbols = CF year 1, grey symbols = AWD year 2, red symbols = CF year 2, grey bar indicates the QTL region as defined by Clump, lines indicate the position of two candidate genes. **b** NJT for the BAAP cultivars for all SNPs located within the Clump region; across the population 71 accessions are in cluster A, 12 in cluster B, 36 in cluster C, and 122 in cluster D. **c** Range of phenotypic variation observed for each cluster across each trait. (Color figure online)

A QTL was detected for grain cadmium in three out of the four experiments (Supplementary Fig. 2) on chromosome 7 between 7.23 and 7.61 Mbp (as defined by Clump). This region revealed QTLs in the studies of Zhao et al. (2018) and Tan et al. (2020). Cluster analysis in this region revealed 4 clusters, with 12 cultivars from cluster B having higher concentrations of grain cadmium (Fig. 4). This 379 kbp region contains 58 gene models, of which 18 are annotated as either transposons or retrotransposons. Of interest in this region are LOC_Os07g12890 (annotated as a metal cation transporter), *OsZIP8* (Zheng et al. 2018), and LOC_Os07g12900 (annotated as a cadmium/zinc-transporting ATPase *OsHMA3*) (Ueno et al. 2010; Sasaki et al. 2014). *OsZIP8* is a group 1 ZIP transporter (Zheng et al. 2018) and has been found to be expressed in the roots and panicles of rice, with expression induced under cadmium stress in seedlings (Zheng et al. 2018). In a previous study (using a mapping population derived from Anjana Dhan and Nipponbare) a QTL for cadmium concentration was mapped to this region of chromosome 7, and *OsZIP8* investigated as a potential candidate gene (Ueno et al. 2010). In that study *OsZIP8* showed no difference in expression in the roots of the parents of the population, and the two different alleles of the genes did not have the ability to transport cadmium in yeast experiments; the authors concluded in that study that *OsZIP8* was not the gene responsible for the differential cadmium accumulation observed in the parents (Ueno et al. 2010).

*OsHMA3* was the first cloned gene involved in cadmium accumulation in rice (Miyadate et al. 2011; Ueno et al. 2010). *OsHMA3* is located on the tonoplast of the root cells and selectively sequesters cadmium into the vacuole, therefore limiting the translocation of cadmium from the roots to the shoots. Studies have identified two natural non-functional alleles of *OsHMA3*; the cultivars with the non-functional alleles have higher cadmium concentrations in the shoots and grains (Ueno et al. 2010; Yan et al. 2016). The first loss-of-function allele was identified in the *indica* accession Anjana Dhan (Ueno et al. 2010). In a field experiment Anjana Dhan was shown to accumulate higher concentrations of cadmium in the shoots and rice grains compared to Nipponbare (*japonica*), a QTL for cadmium concentration was mapped to a 1.9 Mbp region on chromosome 7 (Ueno et al. 2009) and the functional gene identified as *OsHMA3* (Ueno et al. 2010). The non-functional allele from Anjana Dhan is attributed to an amino acid substitution at the 80^th^ position, changing a His to an Arg (Ueno et al. 2010). When examining this polymorphism in the 3000 rice genome accessions on the SNP-Seek database (www.snpseek.irri.org) (Mansueto et al. 2017; Wang et al. 2018), none of the sequenced *aus* accessions have this polymorphism, and neither do any of the *aus* accessions in the BAAP. To date only one *aus* cultivar has been observed to have this polymorphism (Yan et al. 2016).

A second loss-of-function allele for *OsHMA3* was identified in a *japonica* cultivar (Yelicanghua), which has a predicted amino acid mutation at the 380^th^ position from Ser to Arg. The haplotype had no cadmium transport activity when the gene was expressed in yeast, and the allele did not complement a known non-functional allele of *OsHMA3* (Yan et al. 2016). Analysis of the SNPs and sequence data indicates that this is not a polymorphism present in the *aus* accessions in the BAAP. This is supported by the findings of Yan et al. (2016), who only observed this polymorphism in seven accessions from the 950 world rice collection (Huang et al. 2012), with all seven belonging to the temperate *Japonica* subgroup. Another “weak” allele of this gene has recently been described by Sun et al. (2019) but that allele is not present in the BAAP nor in any of the 201 *aus* cultivar in the 3000 rice genome accessions but rather is virtually completely confined to *tropical* and *subtropical japonicas* (based on the 3000 genomes data).

While the *aus* accessions in this population do not have any of the known loss-of-function alleles, *OsHMA3* is still a good candidate gene for this association as both the studies that previously identified the loss-of-function alleles (Ueno et al. 2010; Yan et al. 2016) point to small changes in the DNA sequence having large impacts on the function of this gene. Within the BAAP accessions there are twelve SNPs within the coding regions of *OsHMA3*, five of which are non-synonymous when compared to the Nipponbare sequence. These non-synonymous polymorphisms should be investigated further to see if any cause a change in protein function, and therefore have function effects similar to the previously identified two loss-of-function alleles.

### QTL on chromosome 7 between 8.93 and 9.04 Mbp

**Fig. 5:**
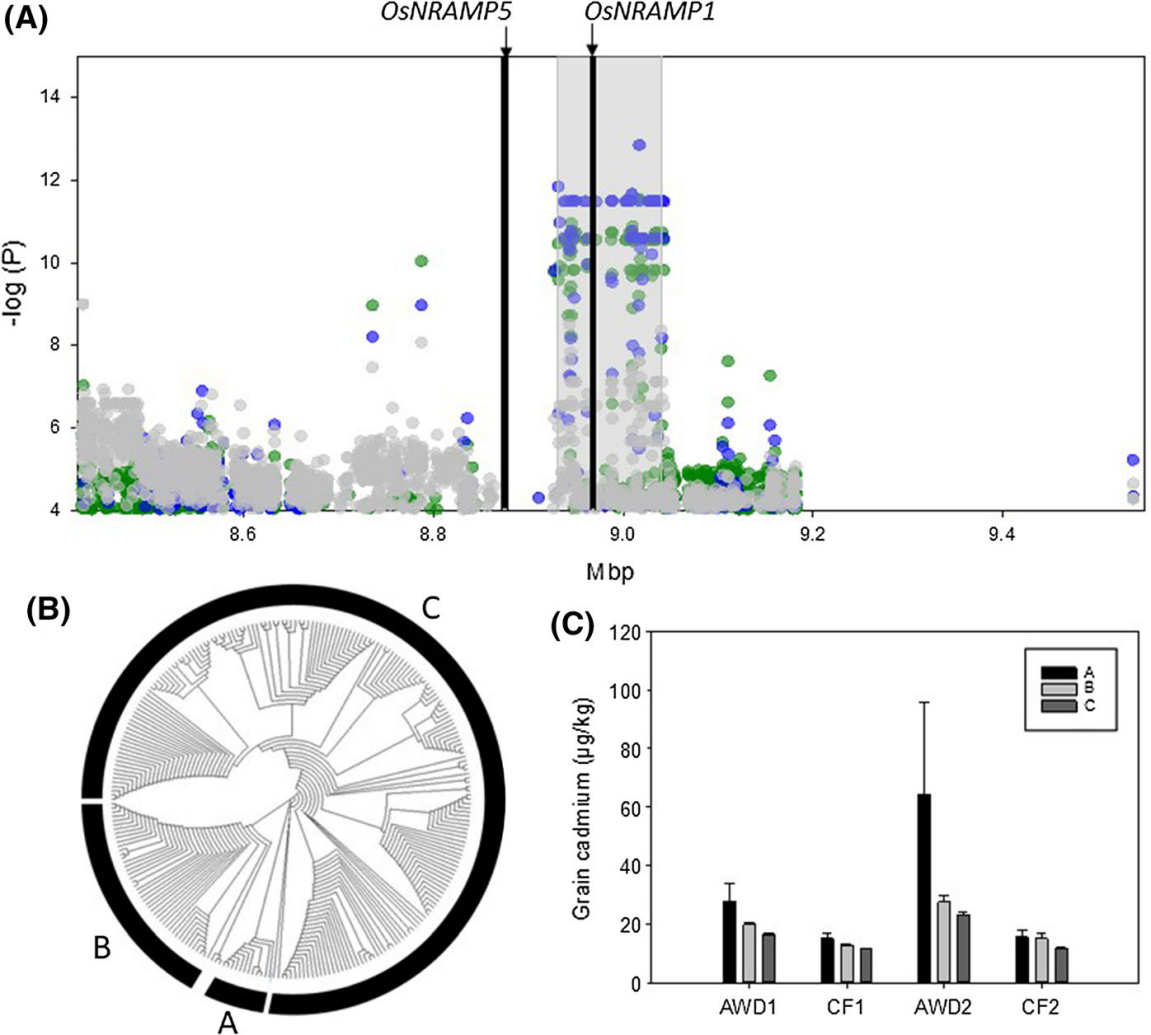
Significant associations for cadmium traits on chromosome 7 (8.4–9.6 Mb). **a** SNPs associated with the cadmium phenotypes; green symbols = AWD year 1, blue symbols = CF year 1, grey symbols = AWD year 2, red symbols = CF year 2, grey bar indicates the QTL region as defined by Clump, lines indicate the position of two candidate genes **b** NJT for the BAAP cultivars for all SNPs located within the Clump region; across the population 7 accessions are in cluster A, 43 in cluster B, and 193 in cluster C. **c** Range of phenotypic variation observed for each cluster across each trait. (Color figure online)

**Fig. 6:**
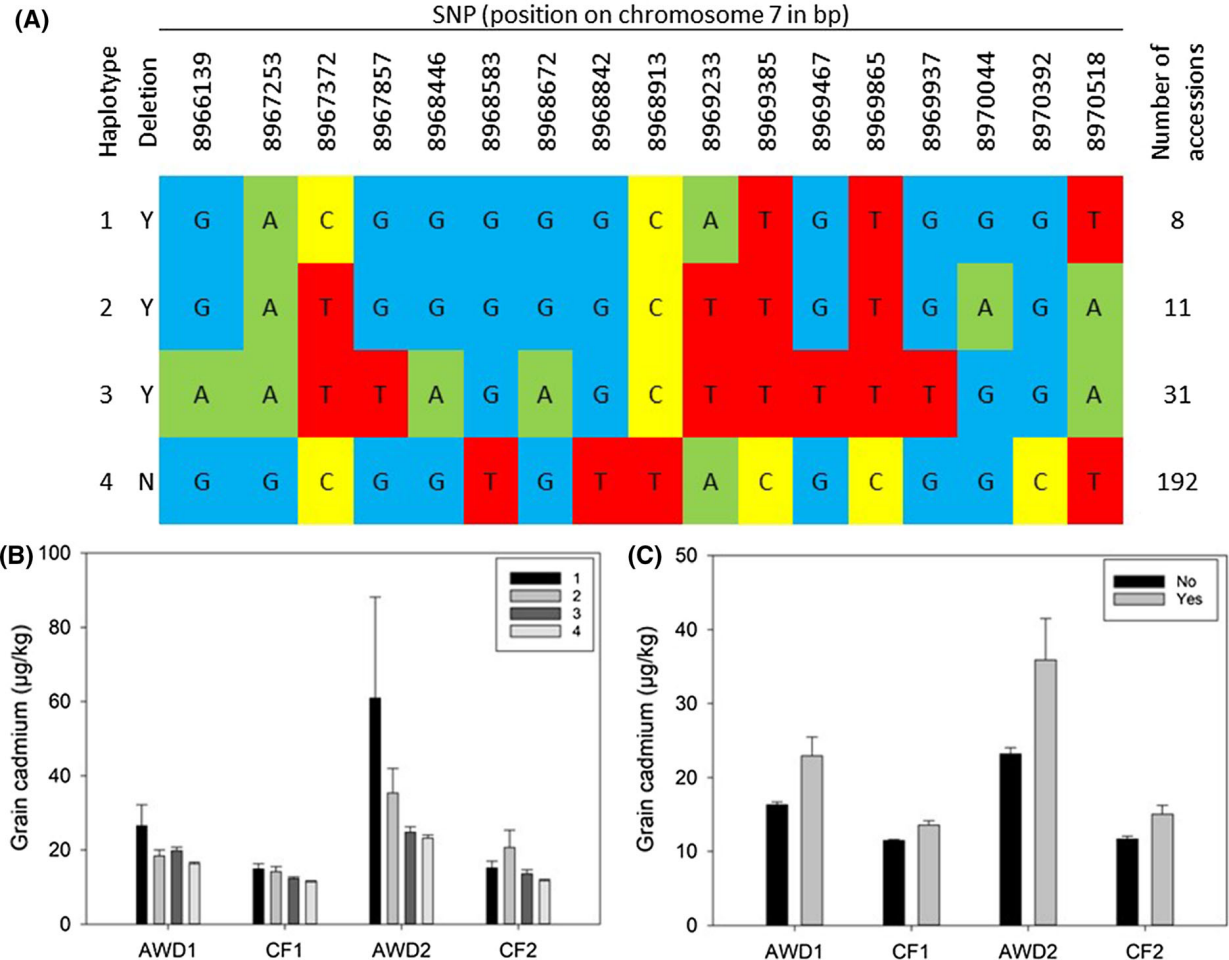
Haplotypes of *NRAMP1* and presence or absence of upstream deletion. **a** Graphical representation of haplotypes, **b** grain cadmium phenotype of the 4 haplotypes of *Nramp1*, **c** grain cadmium phenotype of the accession with and without the deletion upstream of *Nramp1*

A QTL was detected for grain cadmium in three out of the four experiments (Fig. 3 and Supplementary Fig. 2) on chromosome 7 between 8.93 and 9.04 Mbp. This region also revealed QTLs in the studies of Ishikawa et al. (2010), Zhao et al. (2018) and Tan et al. (2020). Cluster analysis in this region revealed 3 clusters with cultivars from cluster A (representing only 7 cultivars) having the highest grain cadmium concentrations (Fig. 5). This 115 kbp region contains 20 gene models, of which 7 are annotated as either transposons or retrotransposons. Within this positional candidate gene list is LOC_Os07g15460, which is annotated as metal transporter Nramp6 and corresponds to *OsNRAMP1* (Mani and Sankaranarayanan 2018). Further analysis of this gene reveals a total of 17 SNPs within *OsNRAMP1* (8,966,023–8,970,882 bp) and these SNPs can be used to classify the accessions into four haplotypes (Fig. 6a). The haplotypes of *OsNRAMP1* correspond to the original clusters generated on SNPs in the whole QTL region: cluster A is haplotype 1, cluster B contains haplotypes 2 and 3, and cluster C is haplotype 4. Out of the 239 accessions where both haplotype and cluster are called, only two accessions don’t follow this pattern: BAAP 25 is assigned to cluster C but has haplotype 3 and BAAP 27 is assigned to cluster B but is haplotype 4. There are significant differences in the cadmium concentrations between the different haplotypes in all four experiments, with the cultivars with haplotype 4 having the lowest grain cadmium concentration (Fig. 6b). It must be noted that haplotype 4 is by some way the most common haplotype in the BAAP population. Visual examination of sequence alignments using the Integrated Genome Viewer (IGV) suggested that some accessions had a deletion upstream of *OsNRAMP1* (8,964,600 to 8,965,003). Further exploration of the sequence read depth around *OsNRAMP1* (see methods) revealed that this deletion is present in all cultivars from haplotypes 1–3, while cultivars from haplotype 4 do not have the deletion. When the phenotype data is analysed based on the presence or absence of the deletion, the cultivars that do not have the deletion have significantly reduced grain cadmium (Fig. 6c). To understand the distribution of this deletion across the diversity of rice the 3000 genome database (Wang et al. 2018) was examined. Using the informative SNPs used to classify the BAAP into the four different haplotypes, the genotypes from the 3000 genome project were classified as haplotypes 1–4 or unknown. Haplotype 1 is only found in *indica* and *aus* type rice, haplotype 2 is dominantly *indica* (94.8%) with a small percentage of *aus* (1.5%) and *japonica* (3.6%), haplotype 3 is only present in *indica* and *aus* rice, and haplotype 4 is most common in *japonica* rice (77.8%) and *aus* (12.4%). These results demonstrate that these haplotypes are predominantly separated based on the *japonica* (haplotype 4) and *indica* (haplotypes 1–3) split in rice, however *aus* cultivars have representation in all haplotypes.This observation of balanced allelic variation only in *aus* highlights why *aus*-based populations like BAAP have the potential to reveal some QTLs that are not revealed in other studies.

Initial cloning of *OsNRAMP1* revealed it was predominantly expressed in the roots (Belouchi et al. 1997), while subsequent analysis demonstrated its role in iron transport (Curie et al. 2000) and its important role in cadmium accumulation in rice (Takahashi et al. 2011). *OsNRAMP1* gene expression is increased in the presence of cadmium and arsenic, with significant differences in gene expression between *indica* and *japonica* accessions (Norton et al. 2008; Zhou et al. 2017). When comparing *indica* and *japonica* rice, the *japonica* rice accession, Azucena, has the complete sequence while the *indica* rice accession, Bala, has the deletion in the upstream region. Based on array data Bala has approximately three times the level of expression of *OsNRAMP1* compared to Azucena in rice roots (Norton et al. 2008). Also, *OsNRAMP1* expression in the roots was higher in a high cadmium-accumulating cultivar (Habataki) than a low cadmium-accumulating cultivar (Sasanishiki) regardless of the presence of cadmium, and the amino acid sequence of *OsNRAMP1* showed 100% identity between Sasanishiki and Habataki (Takahashi et al. 2011). Finally, over-expression of *OsNRAMP1* in rice increased cadmium accumulation in the leaves (Takahashi et al. 2011).

However, it must be noted that this QTL is in close proximity to another gene involved in cadmium accumulation in rice: Os07g15370, *OsNRAMP5* (8.87 Mbp). It has been demonstrated that mutational disruption of *OsNRAMP5* dramatically reduces cadmium in rice grains (Ishikawa et al. 2012). However, this gene is not within the candidate region identified by the Clump analysis (Fig. 5) so in this study *OsNRAMP5* is not a candidate gene for this QTL.

### Other loci with three colocalised QTLs

**Fig. 7:**
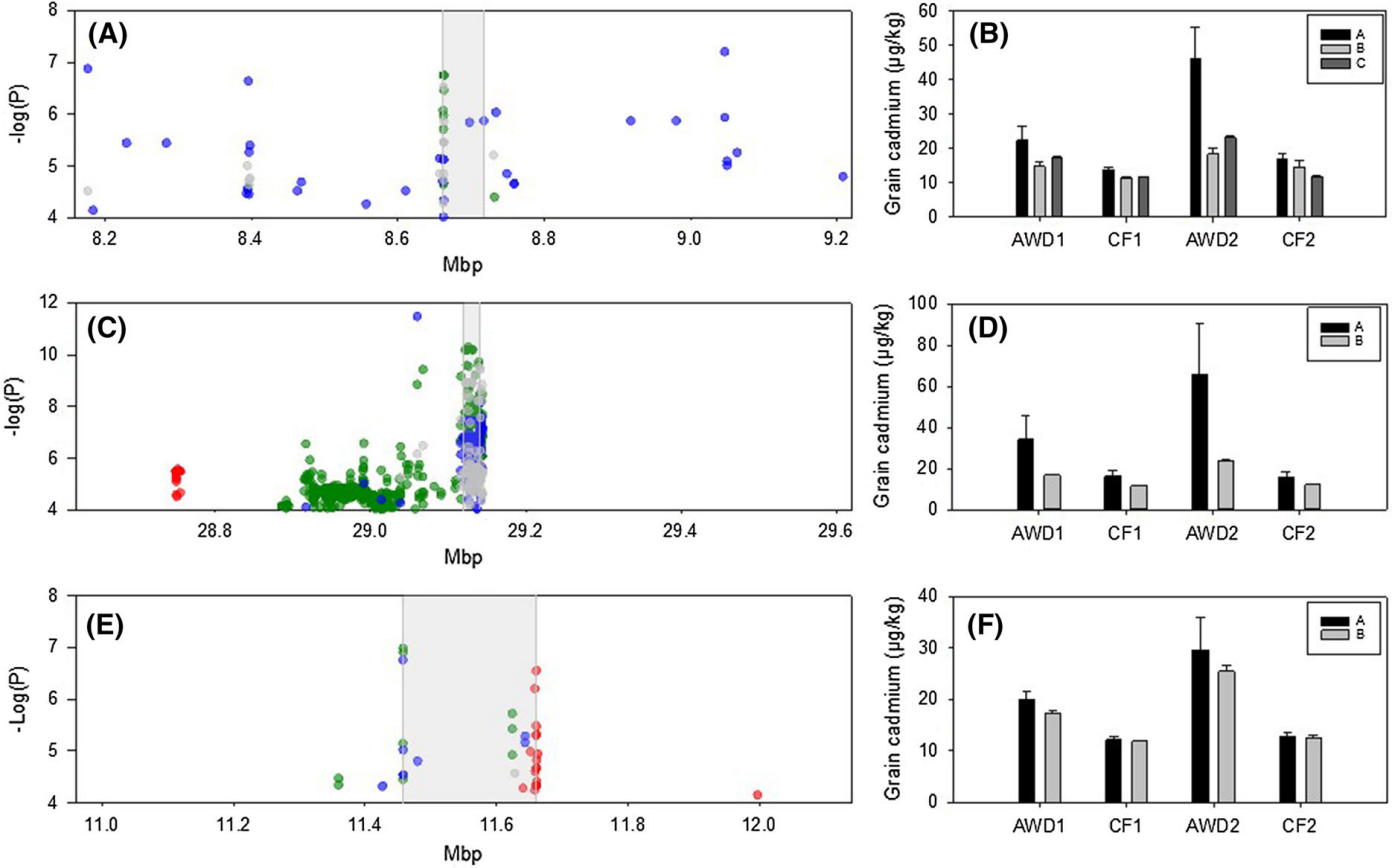
Significant associations for cadmium traits on three genomic positions. **a** Chromosome 5 (8.18–9.22 Mb), **c** chromosome 7 (28.6–29.6 Mb), **e** chromosome 9 (11.0–12.2 Mb); SNPs associated with the arsenic phenotypes; green symbols = AWD year 1, blue symbols = CF year 1, black symbols = AWD year 2, red symbols = CF year 2. Range of phenotypic variation observed for each cluster across each trait. **b** Chromosome 5 (8.18–9.22 Mb) across the population 34 accessions are in cluster A, 8 in cluster B, and 203 in cluster C. **d** chromosome 7 (28.6–29.6 Mb) across the population 12 accessions are in cluster A, and 233 in cluster B. **f** chromosome 9 (11.0–12.2 Mb) across the population 47 accessions are in cluster A, and 200 in cluster B. (Color figure online)

A QTL was detected for grain cadmium in three out of the four experiments (Fig. 7a) on chromosome 5 between 8.66 and 8.72 Mbp. This region has not been reported in other studies. Cluster analysis in this region releveled 3 clusters, with cultivars from cluster A having the highest grain cadmium concentrations. This 55.1 kbp region contains 16 genes (9 annotated as either transposons or retrotransposons), however based on current annotation there are no obvious candidate genes for this locus. A QTL was detected for grain cadmium in three out of the four experiments (Fig. 7b) on chromosome 7 between 29.12 and 29.14 Mbp. This region has not been reported in other studies. Cluster analysis in this region revealed 2 clusters, with cultivars from cluster B (the most common cluster) having the lowest grain cadmium concentrations. This 28.8 kbp region contains 4 genes, however based on current annotation there are no obvious candidate genes for this locus. A QTL was detected for grain cadmium in three out of the four experiments (Fig. 7c) on chromosome 9 between 11.46 and 11.64 Mbp. This region has not been reported in other studies. Cluster analysis in this region revealed 2 clusters, however there was no significant difference in grain cadmium concentration between the clusters. This 202.2 kbp region contains 29 genes (11 annotated as either transposons or retrotransposons), however based on current annotation there are no obvious candidate genes for this locus.

This study has demonstrated the complex genetic regulation for the accumulation of cadmium in rice grains. While a genotype by treatment interaction was observed, there were no clear QTLs that corresponded to cadmium concentration for plants grown under AWD compared to those grown under CF. This could be due to the genotype by treatment interaction being driven by a small number of accessions (i.e. those that are distant from the 1:1 line in Fig. 2), however as this number is small it may mean that the criteria used in the GWA mapping cannot detect the potential low frequency alleles responsible for the phenotype. This low power to detect rare alleles (unless the population is large) is a known limitation of GWA studies (Norton et al. 2014).

## Conclusion

For grain cadmium concentration five QTLs were detected in three of the four experiments. When exploring the contribution of the different genetic clusters around QTLs to the observed phenotype, it must be noted that in a majority of cases the rare cluster contained the accession with high grain cadmium, meaning that a majority of the tested accessions already had the low grain cadmium alleles. Therefore, for breeding, the important consideration will be to ensure that the high cadmium alleles are selected out. Two of these QTLs are at genes known to be involved in cadmium accumulation. While neither of the previously characterised non-functional alleles of *OsHMA3* were observed in the population, there are a number of sequence polymorphisms worthy of further analysis. Additionally, *OsNRAMP1* appears to be an excellent candidate gene for grain cadmium accumulation in this population, not least since many accessions have a large deletion in the promoter associated with higher cadmium. Finally, the impact that water management has on grain cadmium concentration must be taken into account in the future when water saving irrigation practices are being implemented. Utilisation of water management practices with selected rice cultivars should overcome this. This is supported by the study of Ishikawa et al. (2016) where a low grain cadmium cultivar (using a cultivar with an artificially mutated *OsNRAMP5* gene) in combination with a water management technique resulted in rice with both reduced arsenic and cadmium. Future work should focus on applying this same approach but utilising natural allelic variation.

## Supplementary Information

Below is the link to the electronic supplementary material.

Supplementary Figure 1 Grain cadmium data for the population (JPG 1782 kb)

Supplementary Figure 2 QTLs based on clump (JPG 248 kb)

Grain cadmium QTLs in other studies (XLSX 27 kb)

Supplementary file4 (XLSX 19 kb)

Supplementary file5 (XLSX 13 kb)

## Data Availability

The 2 M SNP genotype data of the BAAP is available as a Project called “BAAP” at the SNP-Seek database (http://snp-seek.irri.org/). The phenotype data used for the GWAS is presented in Supplementary Table 1.
