# Supplementary figures and images for: Genetic loci regulating cadmium content in rice grains
Source: Euphytica. 2021 Feb 10;217(3):35. doi: 10.1007/s10681-020-02752-1 (PMC7875855; doi:10.1007/s10681-020-02752-1)

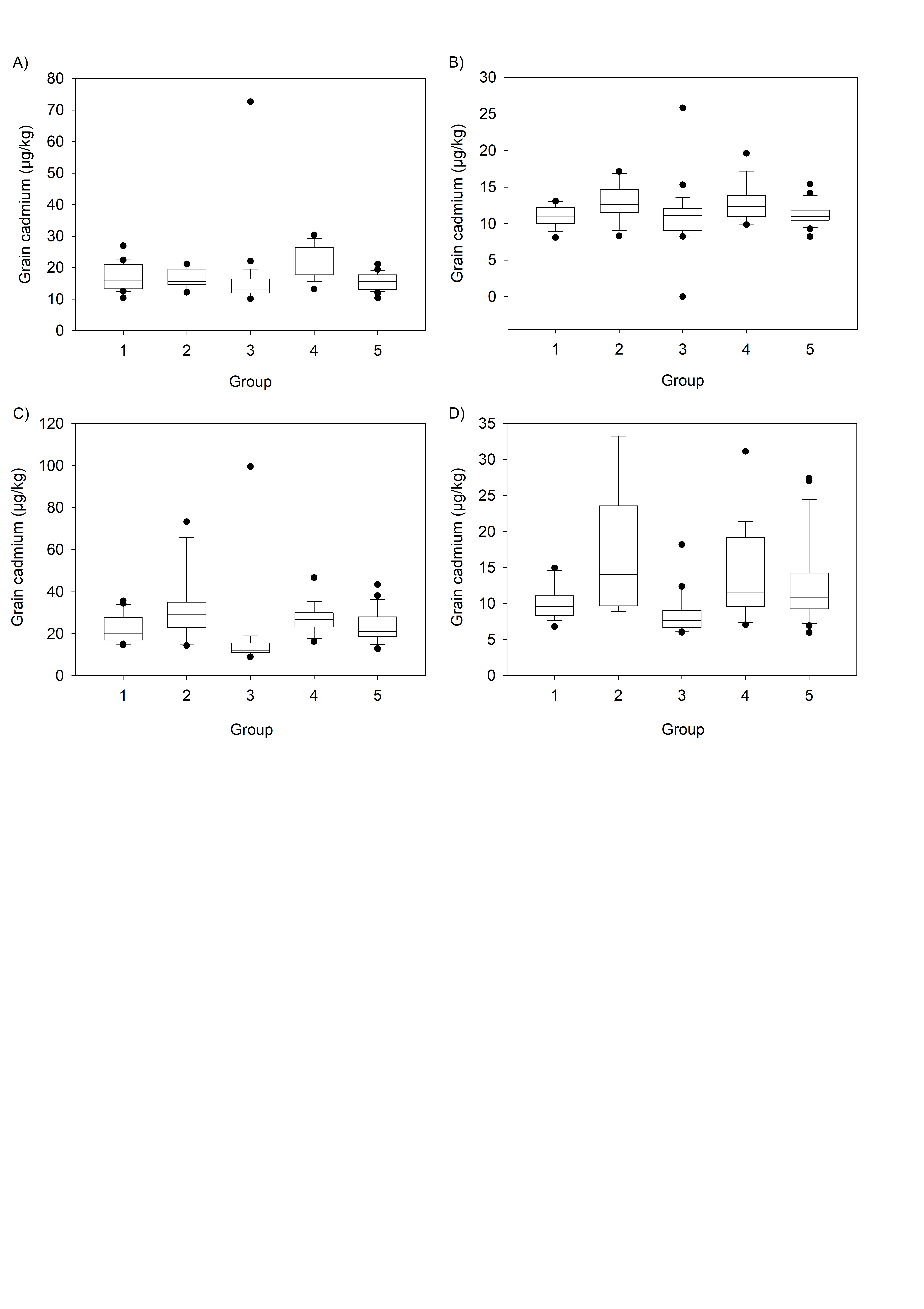

Supplement: Supplementary file 1 — Grain cadmium data for the population (JPG 1782 kb) [file 10681_2020_2752_MOESM1_ESM.jpg]

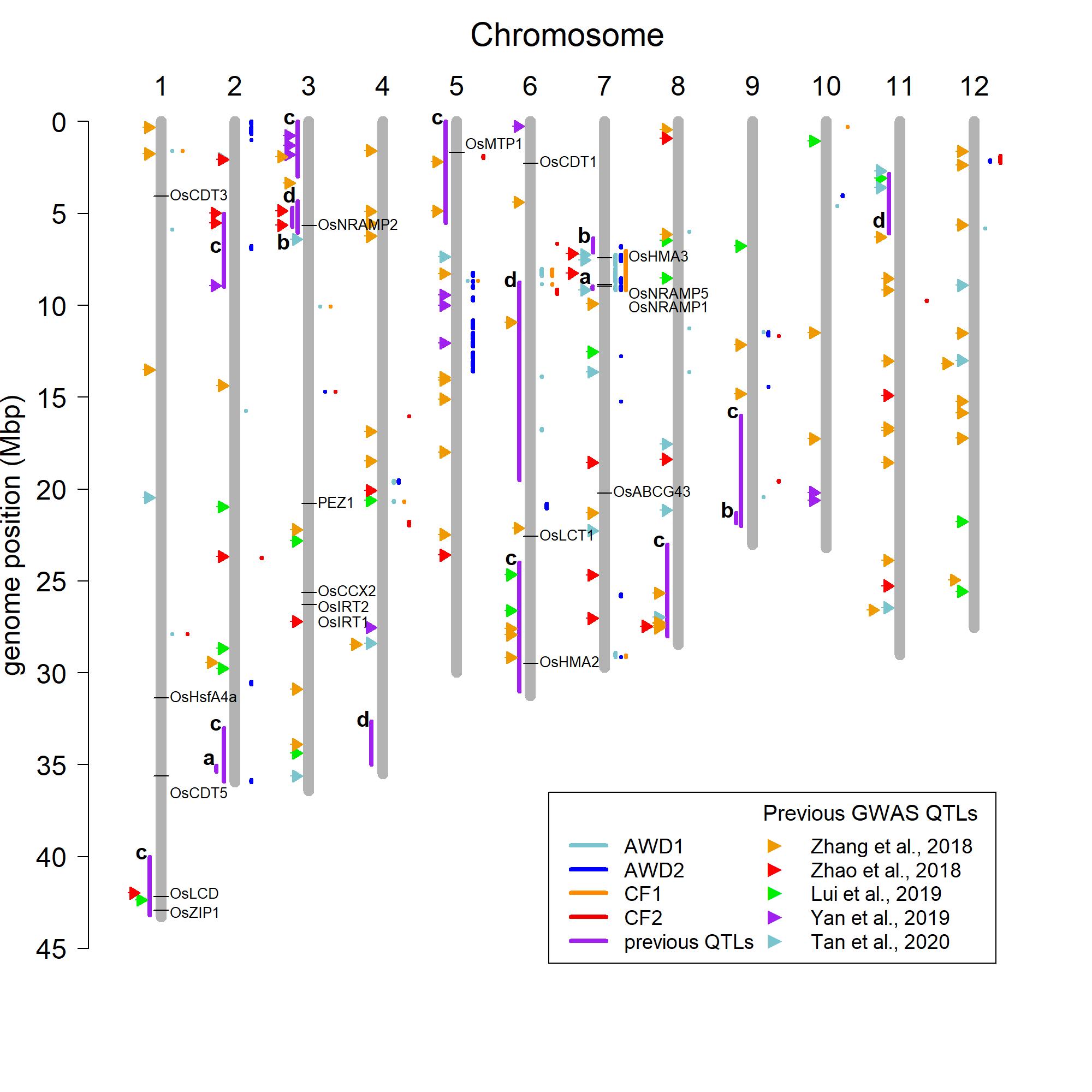

Supplement: Supplementary file 2 — QTLs based on clump (JPG 248 kb) [file 10681_2020_2752_MOESM2_ESM.jpg]
